# Diversity and Functional Analysis of Gut Microbiota in the Adult of *Lissorhoptrus oryzophilus* (Coleoptera: Curculionidae) by Metagenome Sequencing

**DOI:** 10.3390/insects16121260

**Published:** 2025-12-11

**Authors:** Jian-Xue Jin, Yu Wang, Gui-Fen Zhang, Zhao-Chun Ye, Bo Liu, Dan-Dan Yao, Zhao-Chun Jiang, Yong-Fu He

**Affiliations:** 1 Guizhou Branch of State Key Laboratory for Biology of Plant Diseases and Insect Pests, Guizhou Provincial Laboratory of Green Technology and Application Engineering of Plant Protection, Guizhou Key Laboratory of Agricultural Biosecurity, The Institute of Plant Protection, Guizhou Academy of Agricultural Sciences, Guiyang 550006, China; 2 State Key Laboratory for Biology of Plant Diseases and Insect Pests, Key Laboratory for Prevention and Control of Invasive Alien Species of Ministry of Agriculture and Rural Affairs, Institute of Plant Protection, Chinese Academy of Agricultural Sciences, Beijing 100193, China; 3 Agricultural Genomics Institution at Shenzhen, Chinese Academy of Agricultural Sciences, Shenzhen 518120, China; 4 College of Biological and Agricultural Sciences (College of Food Science and Technology), Zunyi Normal University, Zunyi 563006, China; 5 Guizhou Plant Protection Station, Guiyang 550001, China

**Keywords:** *Lissorhoptrus oryzophilus*, gut microbiota, metagenome, diversity analysis, function analysis

## Abstract

The gut microbiota of insects plays a fundamental role in modulating host physiology, including nutrition, development, and adaptability to environmental challenges. The rice water weevil, *Lissorhoptrus oryzophilus* Kuschel (Coleoptera: Curculionidae), is a major invasive pest of rice worldwide, yet the composition and functional profile of its gut microbial community remain poorly characterized. Here, we employed metagenome sequencing on the Illumina NovaSeq X Plus platform to explore the gut microbial diversity and predicted functions in adults of *L. oryzophilus*. Our results revealed a rich microbial community, comprising 26 phyla, 42 classes, 72 orders, 111 families, and 191 genera. The bacterial microbiota was overwhelmingly dominated by the phylum Proteobacteria (85.13% of total abundance). At the genus level, *Pantoea* (48.86%) was the most predominant taxon, followed by *Wolbachia* (14.57%) and *Rickettsia* (11.81%). KEGG analysis suggested that the gut microbiota is primarily associated with metabolic pathways such as membrane transport, carbohydrate and amino acid metabolism, cofactor and vitamin metabolism, energy metabolism, and signal transduction. eggNOG annotation further highlighted significant gene representation in amino acid and carbohydrate transport and metabolism, while CAZy annotation revealed glycosyl transferases (GTs) and glycoside hydrolases (GHs) as the dominant carbohydrate-active enzymes. This study provides the first comprehensive insight into the gut microbiome of *L. oryzophilus* adults, highlighting its potential role in the ecological success of this invasive pest. Our findings lay groundwork for future research aimed at developing novel microbial-based strategies for the sustainable management of *L. oryzophilus*.

## Simple Summary

The rice water weevil, *Lissorhoptrus oryzophilus*, is a highly destructive invasive pest of rice. Its gut hosts a complex community of microorganisms, which may play a crucial role in its adaptation and spread. In this study, we used metagenomic sequencing to comprehensively analyze the structure and potential functions of the gut microbiota in adult weevils. We found that the bacterial community was dominated by the phylum Proteobacteria, with the genus *Pantoea* being most abundant. Functional predictions indicate that these gut microbes are primarily involved in various metabolic processes, such as the digestion of carbohydrates and amino acids, and the breakdown of foreign chemicals. Among the CAZymes identified, glycosyl transferases (GTs) and glycoside hydrolases (GHs) were the most abundant classes. These findings suggest that the gut microbiota likely contributes significantly to the host’s nutrient acquisition and environmental adaptability, providing new insights into the ecological success of this invasive pest.

## 1. Introduction

The rice water weevil, *Lissorhoptrus oryzophilus* Kuschel (Coleoptera: Curculionidae), is one of the most destructive invasive pests native to North America and has been introduced into Asia—the world’s largest rice-producing region [1,2]. In China, it has been successively included in major administrative catalogs, such as the Invasive Alien Species List in China (Second Batch) (2010) [3], the National Key Managed Invasive Alien Species List (First Batch) (2013) [4], and more recently, the Catalogue of Key Managed Invasive Alien Species (2022) [5]. Adults of *L. oryzophilus* exhibit migratory behavior and cause characteristic damage by scraping mesophyll tissue from rice leaves, leaving behind elongated white streaks that significantly impair photosynthesis under high pest densities [6,7]. More critically, the larval stages feed on rice roots, inhibiting root development and leading to plant dislodging and “floating seedlings”, which can result in yield losses ranging from 10% to 25%, and up to 70% in severe cases [8,9,10]. So, this pest poses a significant threat to agricultural production safety in China and other rice-cultivating countries [11]. Effective management of this pest is therefore crucial for agricultural security in rice-growing regions.

There are a wide variety and large number of microorganisms in the insect gut. The insect gut microbiotas form complex symbiotic relationships with their hosts, playing vital roles in nutrition, development, defense, and environmental adaptation [12]. During long-term evolution and adaptation, these microbial communities exhibit remarkable diversity across insect species while maintaining specificity and stability within particular gut niches of a given host [13]. Their functions extend to nutrient metabolism (e.g., degradation of complex plant polymers like cellulose and xylan) and enhancement of host immune responses, thereby influencing the host’s ecological success and invasive potential [14,15,16]. For instance, gut microbes in *Brontispa longissima* enable it to digest cellulose-rich palms, facilitating their adaptation in new environments [17]. Similarly, specific bacteria in *Spodoptera litura* contribute to cellulose and phenol degradation [18]. Thus, the success of insects in their diversification and evolutionary journey has benefited to some extent from the synergistic contributions of gut microbes.

Research on the intestinal symbiotic bacteria of *L. oryzophilus* began in the mid-1990s. Previous research on the gut microbiota has primarily focused on bacterial communities, often relying on culture-dependent methods or 16S rRNA amplicon sequencing. Early studies highlighted the role of *Wolbachia* in the parthenogenesis of non-native populations, with antibiotic treatment confirming its necessity for oocyte production [19]. Subsequent culture-based and 16S rRNA gene surveys reported a relatively low bacterial diversity, dominated by Gammaproteobacteria (e.g., *Pantoea*) and Bacilli, with noted variations across geographical regions [20,21]. However, these approaches provide a limited resolution of the complete microbial community and its functional repertoire.

Advances in metagenomics have revolutionized the study of insect–microbe interactions by enabling comprehensive analysis of microbial communities without the limitations of culturing [22]. Macro-genomics is a microbiological research tool that takes the genes of all microorganisms contained in a specific environment as the object of study, and the relationship between the quantitative structure and function of the microbial flora and the existence of the external environment as the purpose of the study [23]. This culture-independent approach allows for in-depth exploration of the structural and functional potential of gut microbiomes under natural conditions, facilitating the discovery of unculturable taxa and their ecological roles [24,25]. The development of macro-genomes has made it possible to deeply mine the big data of intestinal flora to reveal the profound ecological and evolutionary laws that are difficult to uncover based on small data, which in turn helps to rationally design relevant application [26]. In view of this, the present study employs a metagenomic sequencing approach to achieve a comprehensive profile of the gut microbial diversity and functional potential in adult *L. oryzophilus*. By constructing a small-fragment library for Illumina high-throughput paired-end sequencing and conducting subsequent bioinformatics analyses, we aimed to delineate the taxonomic composition and gene functional capacity of the gut microbiome. Our findings provide a foundational understanding of the microbial involvement in the physiology and invasion ecology of *L. oryzophilus*, offering insights for developing novel, microbiome-based strategies for the sustainable management of this pest.

## 2. Materials and Methods

### 2.1. Species Collection

**Figure 1:**
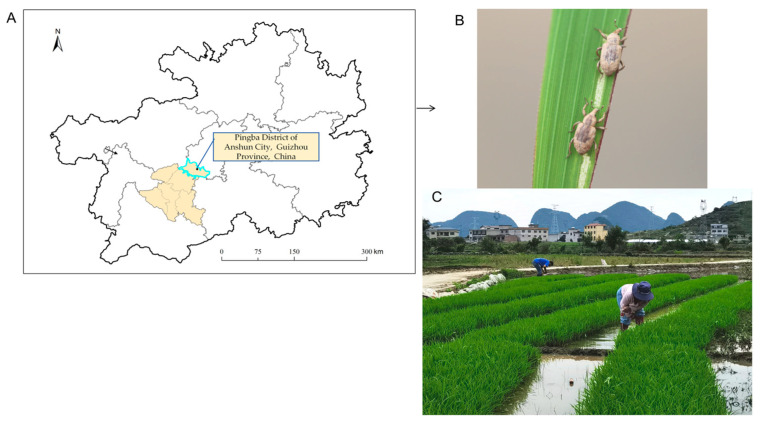
Geographic location of the collection site for *L*. *oryzophilus* adults in their natural habitat. (**A**) Map showing the collection site; (**B**) Adults feed on rice leaves; (**C**) Searching for adults of *L. oryzophilus* in rice seedling fields located in Pingba District of Anshun City, Guizhou Province, China.

Overwintering adults of *L*. *oryzophilus* were manually collected from rice seedlings in a nursery field located in Pingba District, Anshun City, Guizhou Province, China (26.4244° N, 106.3303° E; altitude 1211 m, Figure 1) in May 2024. Pingba is the initial area of *L. oryzophilus* infestation in Guizhou. Collection was conducted during the seedling stage when overwintering adults aggregate on rice seedlings for feeding, which facilitates efficient sampling. The natural occurrence period of the adults in this region spans from May to July, coinciding with optimal environmental conditions and the availability of rice plants for adult nutrition and oviposition.

Following collection, live adults were transferred to the laboratory and maintained in rearing cages (30 cm × 30 cm × 30 cm) at room temperature. They were provided with fresh, indoor-cultured rice seedlings for one day. The roots of the rice seedlings were wrapped in moist absorbent cotton to maintain humidity and placed in a water-filled container.

### 2.2. Gut Dissection

Approximately 1000 overwintering adults starved for 24 h to clear gut contents. Prior to dissection, all dissecting forceps and needles were sterilized with 100% ethanol (Tianjin Fuyu Fine Chenmical Co., Ltd., Tianjin, China), and phosphate-buffered saline (PBS, 0.01 M, pH 7.4; Shenzhen Mohong Technology Co., Ltd., Shenzhen, China) was pre-cooled to 0 °C. The adults were subsequently surface sterilized by three consecutive rinses in 100% ethanol.

The dissection procedure was performed as follows. A droplet of pre-cooled PBS was placed on a sterile Petri dish using a pipette. Individual adults were immersed in the PBS droplet. Under a stereomicroscope (Chongqing COLC Industial Co., Ltd., Chongqing, China), the abdomen was carefully opened with dissecting forceps to expose the internal organs. The intact midgut was then gently separated and transferred into a sterile microcentrifuge tube containing pre-cooled PBS. Throughout the process, all samples were maintained on ice to preserve nucleic acid integrity.

Each biological replicate consisted of pooled midguts from 200 adults. A total of five independent biological replicates (designated PB1 to PB5) were prepared. The samples were immediately flash-frozen in liquid nitrogen and stored at −80 °C until further processing. All samples were subsequently sent to Wuhan Generead Biotechnologies Co., Ltd. (Wuhan, China) for metagenomic DNA extraction, sequencing, and subsequent bioinformatic analysis.

### 2.3. DNA Extraction of Gut Microbiota

Metagenomic DNA was extracted from the gut samples using a standard cetyl trimethyl ammonium bromide (CTAB) protocol (Beijing Solarbio Science & Technology Co., Ltd., Beijing, China). Briefly, the samples were homogenized in 2% (*w*/*v*) CTAB solution and incubated at 65 °C for 1 h. An equal volume of phenol:chloroform:isoamyl alcohol (25:24:1) (Beijing Solarbio Science & Technology Co., Ltd., Beijing, China) was then added for purification. After centrifugation at 12,000× *g* for 10 min at 4 °C, the upper aqueous phase was transferred to a new tube.

Nucleic acids were precipitated by adding an equal volume of isopropanol, incubating for 10 min at room temperature, and centrifuging at 12,000× *g* for 10 min at 4 °C. The resulting DNA pellet was washed twice with 75% (*v*/*v*) ethanol, air-dried, and resuspended in 50 μL of TE buffer containing RNase A (Beijing Solarbio Science & Technology Co., Ltd., Beijing, China), followed by incubation at 37 °C for 30 min to remove RNA contamination. DNA concentration and purity were assessed using a NanoDrop spectrophotometer (Thermo Fisher Scientific, Waltham, MA, USA) by measuring the absorbance ratios at 260/280 nm and 260/230 nm. DNA integrity was further verified by 1.0% agarose gel electrophoresis.

### 2.4. Metagenome Sequencing and Bioinformatic Analysis

Metagenomic sequencing procedure included DNA quality assessment, library construction, library quality control, and high-throughput sequencing on the Illumina NovaSeq X Plus platform (Illumina, San Diego, CA, USA). Raw sequencing reads were first processed to remove adapters and low-quality bases using Trimmomatic (v 0.39) with default parameters to obtain high-quality clean reads. To ensure the accuracy and reliability of downstream analyses, raw sequencing reads were subjected to a stringent quality control (QC) process. Initially, Trimmomatic was employed to filter the raw tags, yielding high-quality clean tags. To remove potential host-derived sequences, the obtained data were aligned against the reference genome of *L. oryzophilus* using Bowtie2 (v 2.5.4), thereby enriching for microbial sequences. The clean readings from each sample were then assembled de novo using MEGAHIT (v1.2.9) to construct contigs and the assembly results were evaluated using the QUAST (v 5.3.0) software. Open reading frames (ORFs) were predicted from the assembled contigs using MetaGeneMark (v 3.26), and the predicted gene sequences were clustered with CD-HIT (v4.6.6) to construct a non-redundant gene catalog.

Taxonomic annotation was performed by aligning the clean reads against the NCBI Non-redundant (Nr) protein database using BLASTP (+2.17.0) (e-value ≤ 1 × 10^−5^). Functional annotation was carried out by aligning the predicted gene sequences against the Kyoto Encyclopedia of Genes and Genomes (KEGG) and Evolutionary Genealogy of Genes: Non-supervised Orthologous Groups (eggNOG). Using HMMER (v 3.0), the protein sequences of the non-redundant genes were compared against the hidden Markov model of each family in the Carbohydrate-active enzymes database (CAZy). Relative species abundance and functional pathway abundance were estimated based on the number of aligned reads.

## 3. Results

### 3.1. Midgut DNA Extraction and Quality Assessment from L. oryzophilus

Genomic DNA was successfully extracted from the midgut of adult *L. oryzophilus*. The concentration and purity of the extracted DNA were evaluated using a nucleic acid protein analyzer, based on the absorbance ratios at OD260/280 and OD260/230. All five samples analyzed exhibited OD260/280 ratios between 2.007 and 2.068, and OD260/230 ratios ranging from 2.356 to 2.436, their sample grade all were “A” (Table 1), indicating high DNA purity. The quality of the extracted DNA met the standards required for downstream metagenomic sequencing analyses.

### 3.2. Quality Control of Gut Microbiota DNA Sequencing Data from L. oryzophilus

Hundreds of thousands of raw tags were generated for each replicate, with the highest number observed in sample PB3 (792,488) and the lowest in PB4 (590,862), reflecting high sequencing depth. After QC, the clean sequence counts were highly consistent with the raw counts, and low-quality sequences accounted for only a minimal proportion. For all replicates, the Q30 values exceeded 98%, Q20 values were above 99%, and the error rate was approximately 0.1%, indicating high sequencing quality and satisfying the standards for reliable metagenomic analysis. The GC content fell within the typical microbial range of 40–60%, supporting the suitability of the data for subsequent genome assembly and annotation (Table 2).

### 3.3. Metagenome Assembly of Gut Microbiota from L. oryzophilus

Metagenome assembly was conducted using MEGAHIT, with contigs shorter than 300 bp being filtered out. In all five replicates, the maximum contig length (Max Len) exceeded 13,000 bp, suggesting the presence of complete or near-complete microbial genomes. The N50 values for each replicate were greater than 1000 bp, and GC content ranged from 48.07% to 53.75%, which is consistent with typical gut microbial communities. These results indicate that the assembly quality was satisfactory for subsequent functional gene annotation and further analysis (Table 3).

**Figure 2:**
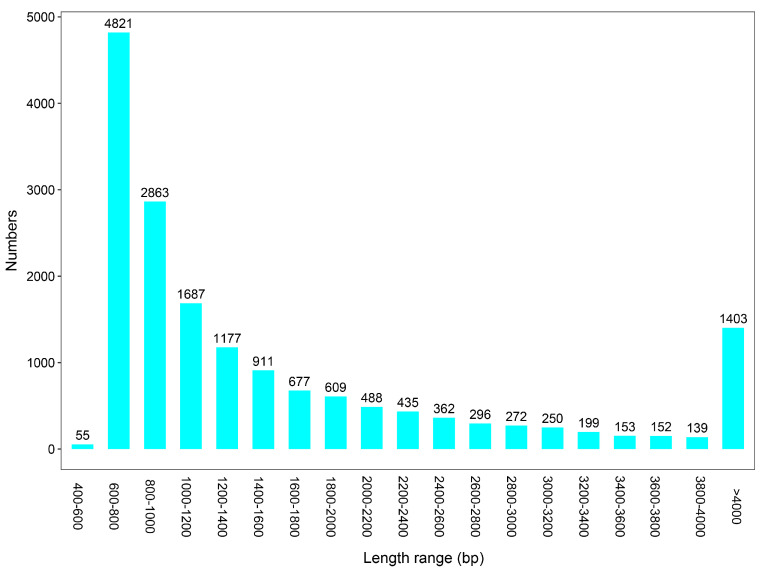
The contig length distribution of gut microbiota from *L. oryzophilus*.

Analysis of contig length distribution revealed that the majority of genes from the gut microbiota of *L. oryzophilus* fell within the 600–800 bp range (4823 contigs), followed by 800–1000 bp (2863 contigs) and 1000–1200 bp (1687 contigs). The number of contigs generally decreased with increasing length; however, a notable number of long contigs (>4000 bp) were still observed (1403 contigs) (Figure 2).

### 3.4. Composition Analysis of Gut Microbiota Metagenome in L. oryzophilus

Gene prediction was performed using MetaGeneMark with default parameters to identify coding regions in the assembled contigs. The predicted number of genes across the five samples ranged from 6493 to 7405, with a GC content of 51.07% to 55.85% (Table 4).

Redundant sequences were subsequently removed with CD-HIT, applying a coverage threshold of 90% and a similarity threshold of 95%. This process yielded a non-redundant gene set of 9053 genes. Among these, the majority of genes (69.09%) contained both start and stop codons, while only 2.04% lacked both. The total length of the predicted genes was 6,366,165 bp, with an average gene length of 703.21 bp. The maximum and minimum gene lengths were 17,046 bp and 102 bp, respectively. The N50 and L50 values were 1020 bp and 2032, and the overall GC content was 51.14%. These metrics indicate high gene integrity and assembly continuity, supporting the suitability of the dataset for downstream functional analyses.

### 3.5. Taxonomic Composition and Relative Abundance of Gut Microbiota in L. oryzophilus

Protein sequences derived from the non-redundant gene set of the gut microbiota were aligned against the Nr database using BLAST. A total of 6879 sequences showed significant matches, enabling taxonomic assignment. Species composition and relative abundance were assessed at five taxonomic levels: phylum, class, order, family, and genus.

Sequences were assigned to three kingdoms: Prokaryota (Archaea and Bacteria), Fungi, and Viruses. At the phylum level, 26 phyla were annotated. Within Archaea, Lokiarchaeota and Thaumarchaeota were identified. Bacterial phyla included Actinobacteria, Aquificae, Bacteroidetes, and Proteobacteria, among others. Fungal phyla comprised Ascomycota, Basidiomycota, and Blastocladiomycota, while viral phyla included Nucleocytoviricota, Negarnaviricota, Peploviricota, and Uroviricota. Proteobacteria was the dominant phylum (85.13%), followed by Chytridiomycota (0.96%), Bacteroidetes (0.29%), and Mucoromycota (0.24%). 12.75% of the total sequences could not be identified at the phylum level.

A total of 42 classes were annotated. Bacterial classes included Actinobacteria, Aquificae, and Bacteroidia; fungal classes comprised Dothideomycetes, Eurotiomycetes, and Leotiomycetes; viral classes included Megaviricetes, Ellioviricetes, Monjiviricetes, Herviviricetes, and Caudoviricetes. Gammaproteobacteria (58.75%) and Alphaproteobacteria (26.66%) were the most abundant classes.

At the order level, 72 orders were identified. These included Nitrosopumilales (Archaea), 32 bacterial orders such as Micrococcales and Streptomycetales, 35 fungal orders (e.g., Mycosphaerellales, Eurotiales), and 5 viral orders (Imitervirales, Bunyavirales, Jingchuvirales, Herpesvirales, Caudovirales). Enterobacterales (55.24%) and Rickettsiales (25.72%) were the dominant orders, followed by Pseudomonadales (3.25%) and Holosporales (1.02%).

In total, 111 families were annotated, including Nitrosopumilaceae (Archaea), 55 bacterial families (e.g., Micrococcaceae, Streptomycetaceae), 58 fungal families (e.g., Mycosphaerellaceae, Periconiaceae), and several viral families such as Mimiviridae and Phenuiviridae. The dominant bacterial families were Erwiniaceae (48.42%), Anaplasmataceae (14.23%), and Rickettsiaceae (11.71%). Yersiniaceae (3.73%), Pseudomonadaceae (3.16%), and Enterobacteriaceae (2.74%) were also notable.

**Figure 3:**
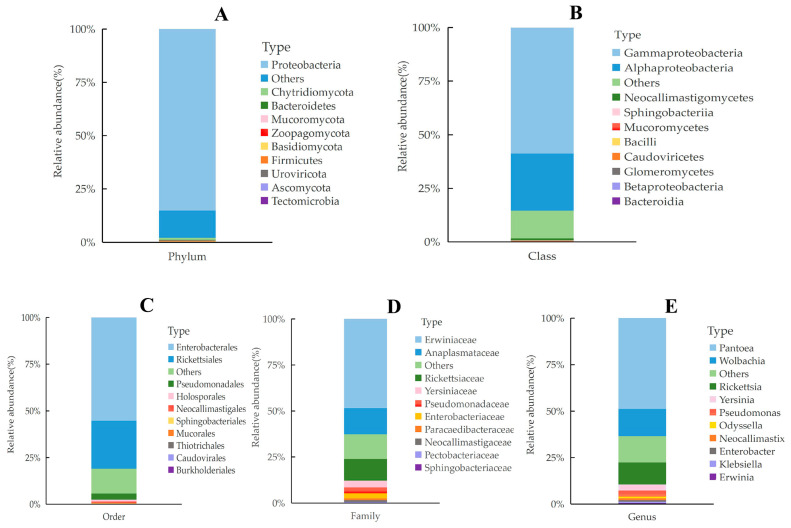
Relative abundance of species at different taxonomic levels of gut microbiota from *L. oryzophilus*. Taxonomic profiles are shown at five hierarchical levels: (**A**) Phylum; (**B**) Class; (**C**) Order; (**D**) Family; (**E**) Genus. Each cell shows the observed percentage of relative abundance for the corresponding taxon.

**Figure 4:**
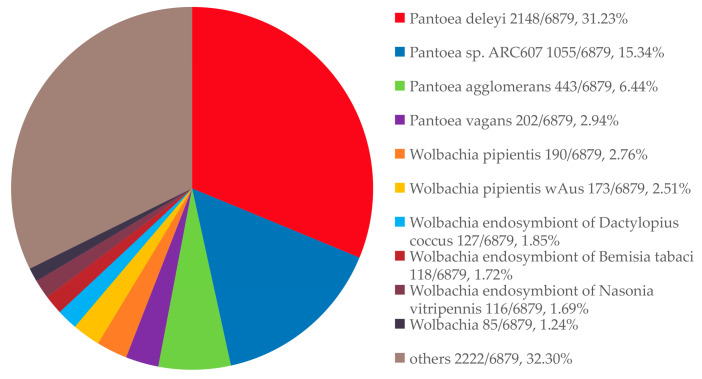
Top 10 species annotated from the Nr database for the gut microbiota of *L. oryzophilus*.

A total of 191 genera were annotated. The most abundant genera were *Pantoea* (48.86%), *Wolbachia* (14.57%), *Rickettsia* (11.81%), *Yersinia* (3.24%), and *Pseudomonas* (3.24%). At the species level, 467 species were identified, with the top 10 all belonging to bacteria. Dominant species included *Pantoea deleyi* (31.23%), *Pantoea* sp. ARC606 (15.34%), *Pantoea agglomerans* (6.44%), *Wolbachia pipientis* (2.76%), and *Wolbachia pipientis* wAus (2.51%). Additionally, 2222 entries were categorized as “others,” accounting for 32.3% of the total annotations (Figure 3 and Figure 4; Table 5).

### 3.6. KEGG Pathway Analysis of Gut Microbiota in L. oryzophilus

**Figure 5:**
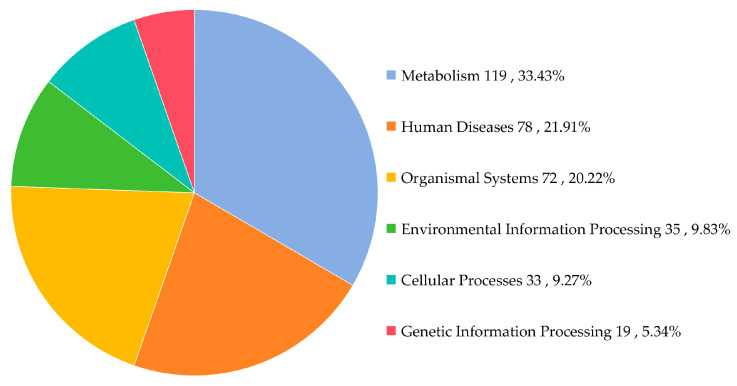
Percentage of primary pathways abundance of gut microbiota in *L. oryzophilus*.

Functional annotation of non-redundant genes from the gut microbiota of *L. oryzophilus* was performed by BLAST alignment against the KEGG database. The relative abundance of pathways at KEGG level 1 and level 2 was assessed. At level 1, six major functional categories were identified: Metabolism (33.43%), Human Diseases (21.91%), Organismal Systems (20.22%), Environmental Information Processing (9.83%), Cellular Processes (9.27%), and Genetic Information Processing (5.34%) (Figure 5). The predominance of metabolic pathways suggests that the gut microbiota primarily contributes to metabolic activities within the host.

**Figure 6:**
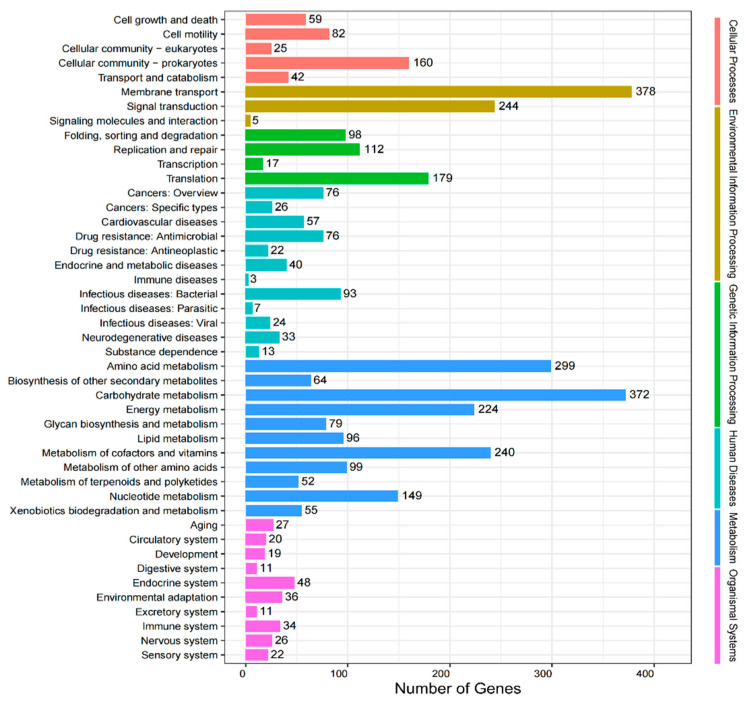
The gene number of KEGG metabolic pathway database analysis of gut microbial secondary functional pathways in *L. oryzophilus*.

A total of 119 level 2 pathways were annotated under Metabolism, followed by Human Diseases (78 pathways), Organismal Systems (72 pathways), Environmental Information Processing (35 pathways), Cellular Processes (33 pathways), and Genetic Information Processing (19 pathways). The pathways with the highest numbers of annotated genes were Membrane Transport (378 genes), Carbohydrate Metabolism (372), Amino Acid Metabolism (299), Signal Transduction (244), and Energy Metabolism (224) (Figure 6).

At the level 2 metabolic pathways, the relative abundances were as follows: Biosynthesis of Other Secondary Metabolites (12.6%), Carbohydrate Metabolism (12.6%), Amino Acid Metabolism (11.7%), Biodegradation and Metabolism of Xenobiotics (11.7%), Metabolism of Cofactors and Vitamins (10.0%), Glycan Biosynthesis and Metabolism (9.2%), Lipid Metabolism (9.2%), Metabolism of Terpenoids and Polyketides (8.4%), Metabolism of Other Amino Acids (6.7%), Energy Metabolism (5.8%), and Nucleotide Metabolism (1.6%). These results indicate that gut microbiota is primarily involved in the metabolism of carbohydrates, amino acids, cofactors, and vitamins, with secondary roles in biodegradation of xenobiotics and biosynthesis of secondary metabolites (Figure 6).

### 3.7. Functional Annotation Based on the eggNOG Database

**Figure 7:**
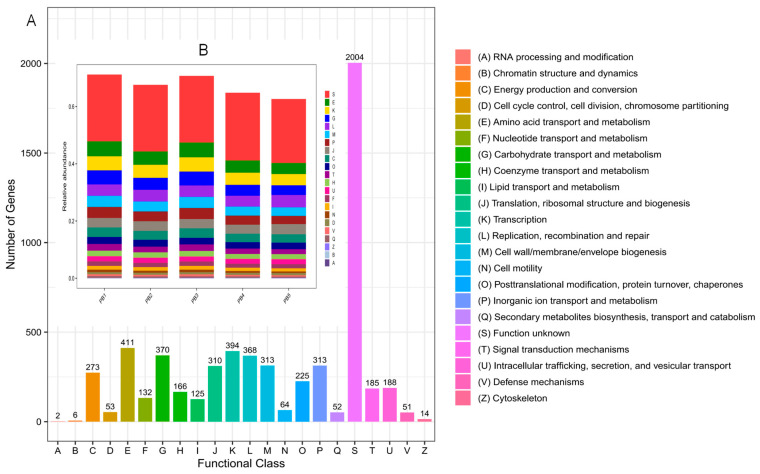
eggNOG annotation of gut microbiota in *L. oryzophilus*. (**A**) eggNOG-based functional classification of the gut microbiota in *L. oryzophilus*; (**B**) The relative abundance of gut microbial categories in *L. oryzophilus* based on eggNOG annotation.

Based on the eggNOG database alignment, the genes from the gut microbiota of *L. oryzophilus* adults were classified into 22 functional categories. The annotation results revealed that the genes encoded by the gut microbiota of *L. oryzophilus* adults were predominantly associated with several core functional categories. These included Amino acid transport and metabolism (411, 6.83%), Transcription (394, 6.55%), Carbohydrate transport and metabolism (370, 6.15%), and Replication, recombination, and repair (368, 6.11%). Other significant categories were Cell wall/membrane/envelope biogenesis (313, 5.20%), and Translation, ribosomal structure and biogenesis (313, 5.15%). Notably, the functions of a substantial proportion of genes (2004, 33.29%) remained uncharacterized (Figure 7).

### 3.8. Functional Annotation Based on the Carbohydrate-Active Enzymes (CAZy) Database

**Figure 8:**
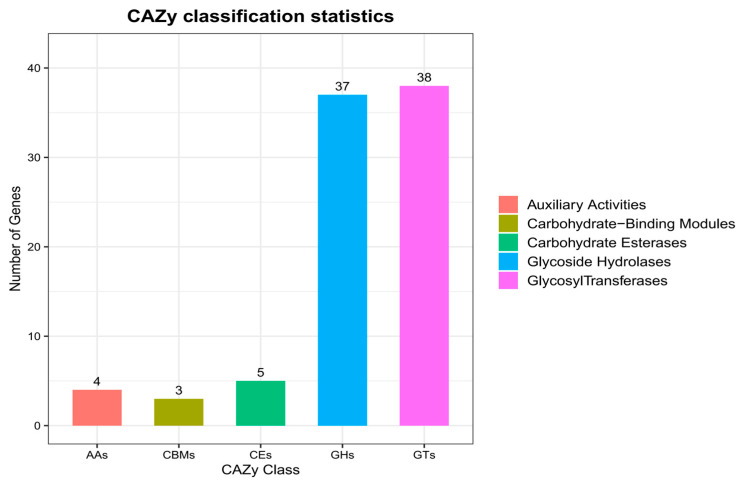
CAZy functional classification of gut microbiota in *L. oryzophilus*.

Based on alignment with the CAZy database, genes from the gut microbiota of *L. oryzophilus* adults were classified into five enzyme classes, with no polysaccharide lyases (PLs) detected. A total of 87 carbohydrate-active enzyme (CAZyme) genes were identified. Among these, 38 genes (43.68%) were annotated as glycosyl transferases (GTs), and 37 genes (42.52%) as glycoside hydrolases (GHs). The percentages of genes annotated as carbohydrate esterases (CEs), auxiliary activities (AAs), and carbohydrate-binding modules (CBMs) were 5.74%, 4.59%, and 3.45%, respectively (Figure 8).

## 4. Discussion

The insect gut serves as a critical site for food storage, digestion, and waste excretion, hosting a diverse and abundant microbial community. Through long-term co-evolution, these microorganisms have established a close mutualistic relationship with their host [27]. The gut microbiota contributes significantly to host physiology by aiding in the digestion and absorption of plant tissues, synthesizing informational compounds and essential nutrients, and enhancing defensive and detoxification capacities. These functions collectively influence key aspects of the host insect’s behavior and life history, including host plant selection, reproduction, and developmental cycles [28,29]. The gut microbiota contributes significantly to host physiology by aiding in the digestion and absorption of plant tissues, synthesizing informational compounds and essential nutrients, and enhancing defensive and detoxification capacities. These functions collectively influence key aspects of the host insect’s behavior and life history, including host plant selection, reproduction, and developmental cycles [30,31].

Metagenomic sequencing enables unbiased characterization of the entire microbial community, capturing non-culturable and obligate anaerobic taxa that frequently dominate insect gut ecosystems—organisms often undetectable by culture-dependent approaches. This method overcomes the limitations of conventional cultivation, which is influenced by the ecological convergence of gut microbiota composition seen in insects with similar feeding habits. In comparison with traditional techniques, metagenomics provides a more comprehensive profile of the gut microbial community, establishing a solid foundation for further research into the mechanisms by which gut microbes influence insect growth, development, and environmental adaptation.

Previous studies have indicated that although the dominant bacterial phyla in the gut microbiota vary across insect species, Proteobacteria consistently represents one of the major groups [32]. For instance, in *Ceracris kiangsu* collected from three geographic locations—Taojiang County (HYT), Xinning County (HSX), and Jiangcheng County (YPJ)—Proteobacteria was the dominant phylum, with relative abundances of 46.7%, 77.0%, and 64.3%, respectively [33]. Similarly, Proteobacteria was reported as the predominant phylum in the larval gut of *Holotrichia parallela* [34]. Phylogenetic analysis of DGGE bands from the intestinal tract of *Atrijuglans hetaohei* larvae showed that 72.5% of the isolated strains belonged to Proteobacteria, while Firmicutes and Bacteroidetes accounted for 12.5% and 15%, respectively. In the leaf-mining beetle *Dactylispa xanthospila*, gut bacteria were classified into 369 genera, 207 families, 135 orders, and 30 phyla, with Proteobacteria (92%), Bacteroidetes (3.4%), and Firmicutes (2.5%) as the dominant groups [35]. In *Nilaparvata lugens*, the dominant gut bacteria also included Proteobacteria, Bacteroidetes, and Firmicutes, while the most abundant fungal phylum was Ascomycota [36]. In contrast, the dominant gut microbes of *Anoplophora glabripennis* were reported as Ascomycota, Firmicutes, Actinobacteria, and Tenericutes [37].

Such compositional differences are also evident at the genus level. For example, in larvae of *Dichocrocis punctiferalis*, 63% of gut bacterial strains belonged to Firmicutes and 37% to Proteobacteria [38]. In the silkworm (*Bombyx mori*), the dominant genera were reported as *Bacillus* and *Arthrospira* [39]. In Chinese populations of *Monochamus alternatus* adults, *Serratia* dominated the midgut microbiota, whereas field-collected adults were primarily colonized by *Enterobacter* [40].

This study provides a comprehensive characterization of the midgut microbiota communities—including Archaea, Bacteria, Fungi, and Viruses—in adult *L*. *oryzophilus*, integrating taxonomic diversity and functional metabolic potential to elucidate the role of gut microbes in host adaptation. Based on metagenomic sequencing, a total of 26 phyla, 42 classes, 72 orders, 111 families, and 191 genera spanning three kingdoms were annotated. Proteobacteria was the dominant phylum (85.13%), followed by the fungal phylum Chytridiomycota (0.96%) and the bacterial phylum Bacteroidetes (0.29%). At the class level, Gammaproteobacteria (58.75%) and Alphaproteobacteria (26.66%) were predominant. Enterobacterales (55.24%) and Rickettsiales (25.72%) represented the dominant orders, while at the genus level, *Pantoea* (48.86%), *Wolbachia* (14.57%), and *Rickettsia* (11.81%) were most abundant.

The bacterium *Pantoea* is a dominant member of the gut microbiota in many insect species, though its functional roles vary across hosts. In some insects, *Pantoea* contributes to the degradation of toxic plant secondary metabolites and the breakdown of complex plant polymers, thereby facilitating nutrient absorption. For example, in *Psylliodes chrysocephala*, which feeds on plants containing isothiocyanates, gut-associated *Pantoea* helps detoxify these compounds and supports the insect’s growth and development [41]. Similarly, phylogenetic analyses have shown that *Zymobacter*, *Arsenophonus*, *Pantoea*, and *Pseudomonas* are dominant in both male and female adults of *Aleurodicus dispersus*, suggesting potential synergistic roles in development, reproduction, and sex-ratio regulation [42].

*Wolbachia*, a maternally inherited endosymbiotic bacterium classified within the order Rickettsiales, is known to induce various reproductive alterations in arthropod hosts [43]. First identified in 1924 in the ovaries of *Culex pipiens* by Hertig and Wolbach, it was formally named *Wolbachia* by Hertig in 1936 [44,45]. This bacterium is widely distributed across numerous insect orders—including Coleoptera, Diptera, Hemiptera, Lepidoptera, Orthoptera, Odonata, Isoptera, and Collembola—and is also present in crustaceans, arachnids, centipedes, and filarial nematodes [45,46,47], Given current estimates of 10–30 million insect species worldwide, it is projected that 15–50 million insect species harbor *Wolbachia*, making it one of the most prevalent symbiotic microorganisms in insects [47]. Taxonomically, *Wolbachia* belongs to Alphaproteobacteria within the phylum Proteobacteria. As maternally inherited intracellular symbiotic bacteria, they infect diverse arthropod hosts, with approximately 70% of insect species in nature estimated to be infected [48]. In recent years, *Wolbachia* has attracted significant research interest due to its ability to induce various reproductive manipulations in host organisms.

Regarding the functional potential of the gut microbiota, previous studies have confirmed that the gut microbial community of *Arsenura armida* possesses key metabolic pathways involved in carbohydrate metabolism, amino acid biosynthesis, and microbial adaptation [49]. The enrichment of glycolysis, gluconeogenesis, and pentose phosphate pathways have also been shown to play important roles in energy production and carbon utilization in termites [50,51]. In the present study, KEGG functional annotation revealed that the gut microbiota of adult *L. oryzophilus* was associated with six level-1 functional categories, with the highest representation in Human Diseases, Metabolism, and Organismal Systems. At level 2, the gut microbiota was primarily involved in metabolic functions. The most abundant level-2 pathways included membrane transport, carbohydrate metabolism, amino acid metabolism, metabolism of cofactors and vitamins, energy metabolism, and signal transduction. Furthermore, genes associated with purine metabolism, ABC transporters, two-component systems, and ribosomes were prominently identified, underscoring the metabolic versatility and adaptive capacity of the gut microbial community in *L. oryzophilus*.

To predict gene function, the protein sequences derived from non-redundant genes were aligned against the eggNOG database using BLAST. The best hit for each sequence was identified, and the corresponding functional and categorical information was assigned to the query gene. The eggNOG database is widely utilized for classifying and annotating genes in newly sequenced genomes, providing information on orthologous groups across various taxonomic levels, including eukaryotes, prokaryotes, and viruses. The CAZy database is a specialized resource for the study of carbohydrate-active enzymes, primarily encompassing six major functional classes: GHs, GTs, PLs, CEs, AAs, and CBMs. All families meeting the filtering threshold were identified, enabling the annotation of carbohydrate-active enzymes and the analysis of their conserved functional domains in the genomic data.

In this study, Functional annotation via eggNOG revealed that a considerable number of genes from the gut microbiota of *L. oryzophilus* adults were associated with categories such as amino acid transport and metabolism and carbohydrate transport and metabolism. This finding was corroborated by KEGG pathway analysis, which showed significant enrichment in carbohydrate metabolism, amino acid metabolism, and metabolism of cofactors and vitamins. Furthermore, alignment against the CAZy database indicated that the predominant Carbohydrate-Active Enzymes were glycosyl transferases (GTs) and glycoside hydrolases (GHs). The consistency across these three databases strongly suggests that a primary function of the gut microbiota in *L. oryzophilus* is metabolism, assisting the host in the digestion and transport of amino acids and carbohydrates. This microbial activity facilitates energy conversion, provides metabolic substrates, and enhances nutrient absorption. Specifically, it enables the weevil to more efficiently digest nutrients like amino acids and carbohydrates present in rice leaves, thereby supporting its normal growth and survival. Additionally, a significant number of genes were categorized under information storage and processing, including functions related to “Transcription”, “Replication, recombination and repair”, “Translation, ribosomal structure and biogenesis”, and “Cell wall/membrane/envelope biogenesis”. These functions are critical for sustaining microbial growth, reproduction, and the normal physiological activities of gut cells. It is noteworthy that 2004 genes were annotated as “function unknown”. The abundance of these uncharacterized genes implies the presence of substantial undiscovered bioinformation. Further investigation into these genes is still warranted.

This study provides initial insights into the community composition and potential functions of the gut microbiota in *L. oryzophilus*, while also confirming the presence of a substantial number of unclassified microbial taxa in this insect’s gut. However, the interactions among these unknown taxa and their effects on host survival and adaptation remain poorly understood. Building on these problems, we will expand systematic sampling of *L. oryzophilus* across different ecological regions and conduct ongoing functional analyses of the gut microbiota, with the aim of providing a scientific basis for the development of effective control strategies. Future research should integrate synthetic microbial community experiments, co-occurrence network analysis, and metabolomics—together with other multi-omics approaches—to further elucidate microbial interactions, functional roles, underlying mechanisms, and the dynamics of community stability within the gut ecosystem of *L. oryzophilus*.

## 5. Conclusions

This study employed Illumina NovaSeq X Plus sequencing to investigate the diversity and functional potential of the gut microbiota in adult *L*. *oryzophilus*. Taxonomic profiling based on the NR database revealed a community comprising 26 phyla, 42 classes, 72 orders, 111 families, and 191 genera, with Proteobacteria being the dominant phylum (85.13%) and *Pantoea* being the most abundant genus. Functional predictions consistently indicated strong metabolic specialization. KEGG analysis highlighted the predominant roles of metabolic pathways, including carbohydrate and amino acid metabolism, xenobiotic biodegradation, secondary metabolite biosynthesis, and genetic information processing. Concordant results from eggNOG annotation showed significant gene abundances related to amino acid and carbohydrate transport and metabolism. Furthermore, CAZy database annotation identified glycosyl transferases (GTs) and glycoside hydrolases (GHs) as the predominant carbohydrate-active enzymes. The remarkable functional consistency across these databases underscores a primary role of the gut microbiota in metabolism, likely assisting the host in nutrient digestion and energy acquisition. These metabolic capabilities may underpin the rapid environmental adaptation and high fitness of *L. oryzophilus*, potentially contributing to its success as an invasive species. Future mechanistic studies are warranted to validate these functional associations and elucidate their ecological implications.

## Figures and Tables

**Table 1:** Intestinal DNA testing of gut microbiota from *L. oryzophilus*.

Serial Number	Sample Name	Qubit Concentration (ng/μL)	Total Amount (μg)	OD260/280	OD260/230	Sample Grade *	Test Result
1	PB1	152.0	5.32	2.062	2.384	A	eligible
2	PB2	148.1	5.19	2.068	2.356	A	eligible
3	PB3	148.9	5.21	2.062	2.416	A	eligible
4	PB4	119.9	4.2	2.007	2.436	A	eligible
5	PB5	147.3	5.16	2.007	2.365	A	eligible

Notes: OD260/280, OD260/230 ratio ideal range respectively: 1.8–2.1, 2.0–2.5. * Sample Grades A: Sample quality meets the requirements for library construction.

**Table 2:** Sample sequencing data evaluation statistics of gut microbiota from *L. oryzophilus*.

Sample	Raw Reads	Raw Bases	Trimmed Reads	Trimmed Bases	Trimmed_Q20	Trimmed_Q30	Trimmed_GC	Effective
PB1	765,994	114,899,100	764,622	112,788,700	99.72%	98.79%	47.00%	98.16%
PB2	675,668	101,350,200	673,830	97,153,533	99.44%	98.17%	45.77%	95.86%
PB3	792,488	118,873,200	790,040	114,780,156	99.40%	98.03%	47.08%	96.56%
PB4	590,862	88,629,300	588,910	85,678,697	99.43%	98.12%	44.10%	96.67%
PB5	603,798	90,569,700	601,938	88,026,543	99.39%	98.00%	43.14%	97.19%

Note: Raw Reads: number of raw sequencing reads; Raw Bases: total number of bases in raw reads; Trimmed Reads: number of reads after quality trimming; Trimmed Bases: total number of bases after quality trimming; Trimmed_Q20: Q20 percentage after quality trimming (%); Trimmed_Q30: Q30 percentage of reads remaining after quality trimming (%); Trimmed_GC: GC content after quality trimming (%); Effective: effective rate of the sequencing data (%).

**Table 3:** Statistics of metagenome assembly results of gut microbiota from *L. oryzophilus*.

Sample	Number	Total Len (bp)	Average Len (bp)	Max Len (bp)	Min Len (bp)	N50	L50	N90	L90	GC (%)
PB1	3141	4,999,651	1591.74	13,320	600	2024	732	753	2394	53.75
PB2	3079	6,522,469	2118.37	19,046	502	3401	543	832	2151	49.68
PB3	2900	7,109,211	2451.45	70,421	507	5740	268	818	1888	49.13
PB4	4036	6,301,918	1561.43	14,834	545	1931	939	756	3108	48.61
PB5	3793	6,012,806	1585.24	27,067	515	1948	836	761	2908	48.07

Note: Sample: Sample ID; Number: number of assembled contigs; Total Len: total length of all contigs (bp); Average Len: average length of contigs (bp); Max Len: length of the longest contig (bp); Min Len: length of the shortest contig (bp); N50: the length of the shortest contig in the set of largest contigs whose combined length represents at least 50% of the total assembly length; L50: the smallest number of contigs whose combined length represents at least 50% of the total assembly length; N90: the length of the shortest contig in the set of largest contigs whose combined length represents at least 90% of the total assembly length; L90: the smallest number of contigs whose combined length represents at least 90% of the total assembly length; GC (%): the percentage of guanine (G) and cytosine (C) bases among the total bases in the assembly.

**Table 4:** Statistical analysis of genetic prediction results of gut microbiota from *L. oryzophilus*.

Sample	Number	Integrity: Start	Integrity: End	Integrity: All	Integrity: None	Total Len (bp)	Average Len (bp)	Max Len (bp)	Min Len (bp)	N50	L50	GC (%)
PB1	6493	1241 (19.11%)	1970 (30.34%)	3056 (47.07%)	226 (3.48%)	3,919,968	603.72	3807	102	831	1599	55.85
PB2	7246	1000 (13.80%)	1657 (22.87%)	4440 (61.28%)	149 (2.06%)	4,924,452	679.61	6732	102	939	1741	52.61
PB3	7189	766 (10.66%)	1279 (17.79%)	5043 (70.15%)	101 (1.40%)	5,225,364	726.86	17,046	102	1011	1707	52.94
PB4	7405	1275 (17.22%)	2215 (29.91%)	3708 (50.07%)	207 (2.80%)	4,449,258	600.85	4905	102	831	1796	52.40
PB5	7282	1238 (17.00%)	2156 (29.61%)	3681 (50.55%)	207 (2.84%)	4,372,515	600.46	5271	102	834	1733	51.07

Note: Sample: sample ID; Number: predicted number of genes; Integrity: proportion of gene integrity (Start: genes with only a start codon; End: genes with only a stop codon; All: genes with both start and stop codons; None: genes lacking both start and stop codons); Total Len: total base pairs of all predicted genes; Average Len: average base pairs of predicted genes; Max Len: length of the longest gene; Min Len: length of the shortest gene; N50: the length of the shortest contig in the set of largest contigs whose combined length represents at least 50% of the total assembly length; L50: the smallest number of contigs whose combined length represents at least 50% of the total assembly length; GC (%): the percentage of guanine (G) and cytosine (C) bases among the total bases in the assembly.

**Table 5:** Diagram of the annotation results of the Nr database of gut microbiota in *L. oryzophilus*.

Kingdom	Phylum	Class	Order	Family	Genus	Species
Bacteria	Proteobacteria	Gammaproteobacteria	Enterobacterales	Erwiniaceae	*Pantoea*	*Pantoea deleyi*
						*Pantoea* sp. ARC607
						*Pantoea agglomerans*
						*Pantoea vagans*
		Alphaproteobacteria	Rickettsiales	Anaplasmataceae	*Wolbachia*	*Wolbachia pipientis*
						*Wolbachia pipientis* wAus
						*Wolbachia endosymbiont* of *Dactylopius coccus*
						*Wolbachia endosymbiont* of *Bemisia tabaci*
						*Wolbachia endosymbiont* of *Nasonia vitripennis*
						*Wolbachia*

## Data Availability

The original contributions presented in this study are included in the article. Further inquiries can be directed to the first author (jinjianxue163@163.com).

## Abbreviations

The following abbreviations are used in this manuscript:

KEGG	Kyoto Encyclopedia of Genes and Genomes
Nr	Non-Redundant Protein Database
eggNOG	Evolutionary Genealogy of Genes: Non-supervised Orthologous Groups
CAZy	Carbohydrate-Active enZymes
GHs	Glycoside hydrolases
GTs	Glycosyl transferases
PLs	Polysaccharide lyases
CEs	Carbohydrate esterases
AAs	Auxiliary activities
CBMs	Carbohydrate-binding modules

## References

[1] Kuschel G. (1951). Revision of Lissorhoptrus of Contey generos vecinos in America. Rev. Chil. Entomol..

[2] Heinrichs E.A., Quisenberry S.S., Clement S.L., Quisenberry S.S. (1999). Germplasm evaluation and utilization for insect resistance in rice. Global Plant Genetic Resources for Insect-Resistance Crops.

[3] Ministry of Ecology and Environment of the People’s Republic of China (2010). Announcement of the Former State Environmental Protection Administration: The Second Batch of Invasive Alien Species in China (Huan Fa [2010] No. 4). https://www.mee.gov.cn/gkml/hbb/bwj/201001/t20100126_184831.htm.

[4] (2013). Announcement No. 1897 of the Ministry of Agriculture of the People’s Republic of China. J. Biosaf..

[5] Ministry of Agriculture and Rural Affairs of the People’s Republic of China (2022). Announcement No. 567: Catalogue of Key Managed Invasive Alien Species. http://www.moa.gov.cn/govpublic/KJJYS/202211/t20221109_6415160.htm.

[6] Heinrichs E.A., Heinrichs E.A. (1994). Host plant resistance. Biology and Management of Rice Insects.

[7] Saito T., Hirai K., Way M.O. (2005). The rice water weevil, *Lissorhoptrus oryzophilus* Kusehel (Coleoptera: Curculionidae). Appl. Entomol. Zool..

[8] Way M.O., Grayson B.T., Green M.B., Copping L.G. (1990). Insect pest management in rice in the United States. Pest Management in Rice.

[9] Zou L.I., Stout M.J., Dundand R.T. (2004). The effects of feeding by the rice water weevil, *Lissorhoptrus oryzophilus* Kushel, on the growth and yield components of rice, *Oryza sativa*. Agric. For. Entomol..

[10] Reay-Jones F.P.F., Way M.O., Tarpley L. (2008). Nitrogen fertilization at the rice panicle differentiation stage to compensate for rice water weevil (Coleoptera: Curculionidae) injury. Crop. Prot..

[11] Gao Y.L., Reitz S.R. (2017). Emerging themes in our understanding of species displacements. Annu. Rev. Entomol..

[12] Macke E., Tasiemski A., Massol F., Callens M., Decaestecker E. (2017). Life history and eco-evolutionary dynamics in light of the gut microbiota. Oikos.

[13] Chen C., Lin J., Lin Z., Li Q.R., Huang J.Y., Wu Q.J., Ji Q.H. (2024). Functions and applications of intestinal symbiotic microorganisms in insects. Asian Agric. Res..

[14] Cui L., Fang Y., Zhou Z.B., He Y.Q., Zhang Y. (2025). Research progress on pathogens carried by sand flies and their gut microbiota. Chin. J. Parasitol. Parasit. Dis..

[15] Hooper L.V., Bry L., Falk P.G., Gordon J.I. (1998). Hosts-microbial symbiosis in the mammalian intestine: Exploring an internal ecosystem. BioEssays.

[16] Omondi Z.N., Arserim S.K., Töz S., Özbel Y. (2022). Host-parasite interactions: Regulation of Leishmania infection in sand fly. Acta Parasitol..

[17] Zhang Y.L., Lyu B.Q., Yang F., Tu Y., Jiang F.Y.D., Qi K.X., Li Z.C. (2021). Isolation, identification and functional analysis of intestinal microorganisms of *Brontispa longissimi* Gestro. Chin. J. Tropic. Crops.

[18] Sun B.T., Lan B.M., Wang Q., Xia X.F., You M.S. (2017). Isolation and Preliminary functional analysis of the larval gut bacteria from *Spodoptera litura* larvae. Biot. Resour..

[19] Chen S.J., Lu F., Jiang M.X., Way M.O. (2012). Identification and biological role of the endosymbionts *Wolbachia* in rice water weevil (Coleoptera: Curculionidae). Environ. Entomol..

[20] Lu F., Kang X., Jiang C., Lou B.G., Jiang M.X., Way M.O. (2013). Isolation and characterization of bacteria from midgut of the rice water weevil (Coleoptera: Curculionidae). Environ. Entomol..

[21] Lu F., Kang X., Lorenz G., Espino L., Jiang M.X., Way M.O. (2014). Culture-independent analysis of bacterial communities in the gut of rice water weevil (Coleoptera: Curculionidae). Ann. Entomol. Soc. Am..

[22] Douglas A.E. (2015). Multiorganismal insects: Diversity and function of resident microorganisms. Annu. Rev. Entomol..

[23] Yu H., Li H. (2022). Research progress in the application of metagenomics in animal gut microbes. Anhui Agric. Sci..

[24] Suau A., Bonnet R., Sutren M., Godon J.J., Gibson G.R., Collins M.D., Doré J. (1999). Direct analysis of genes encoding 16S rRNA from complex communities reveals many novel molecular species within the human gut. Appl. Environ. Microb..

[25] Furrie E. (2006). A molecular revolution in the study of intestinal microflora. Gut.

[26] Cao L., Ning K. (2018). Metagenomics of insect gut: New borders of microbial big data. Acta Microbiol. Sin..

[27] Engel P., Moran N.A. (2013). The gut microbiota of insects-diversity in structure and function. FEMS Microbiol. Rev..

[28] Fischer C.N., Trautman E.P., Crawford J.M., Stabb E.V., Handelsman J.O., Broderick N.A. (2017). Metabolite exchange between microbiome members produces compounds that influence *Drosophila* behavior. Elife.

[29] Zhou F., Pang Z.C., Yu X.Q., Wang X.Y. (2020). Insect gut microbiota research: Progress and applications. Chin. J. Appl. Entomol..

[30] Lu Y.X., Liu Y.Q., Li Q., Xia R.X., Wang H. (2016). Research progress on intestinal microbial diversity of insects. J. Henan Agric. Sci..

[31] Cheng D.F., Li H.J., Lu Y.Y. (2021). Research progress of the influence of microorganisms on insect behavior. Acta Entomol. Sin..

[32] Ji-Hyun Y., Woon S.R., Woong T.W., Jung M.J., Kim M.S., Park D.S., Yoon C.M., Nam Y.D., Kim Y.J., Choi J.H. (2014). Insect gut bacterial diversity determined by environmental habitat, diet, developmental stage, and phylogeny of host. Appl. Environ. Microb..

[33] Yang L.J., Li H.W., Li X.Y., Shen A.D., Yu Y.X. (2023). Diversity analysis of gut microbes of yellow spined bamboo locust *Ceracris kiangsu* Tsai. J. Environ. Entomol..

[34] Huang S.W., Zhang H.Y. (2017). The impact of environmental heterogeneity and life stage on the hindgut microbiota of *Holotrichia parallela* larvae (Coleoptera: Scarabaeidae). PLoS ONE.

[35] Cui L.X., Guo Q.Y., Wang X.X., Duffy K.J., Dai X.H. (2021). Midgut bacterial diversity of a leaf-mining beetle, *Dactylispa xanthospila* (Gestro) (Coleoptera: Chrysomelidae: Cassidinae). Biodivers. Data J..

[36] Wang T.Z., Wang Z.L., Zhu H.F., Wang Z.Y., Yu X.P. (2019). Analysis of the gut microbial diversity of the brown planthopper, *Nilaparvata lugens* (Hemiptera: Delphacidae) by high-throughput sequencing. Acta Entomol. Sin..

[37] Schloss D.P., Delalibera I., Handelsman J., Raffa K.F. (2006). Bacteria associated with the guts of two wood-boring beetles: *Anoplophora glabripennis* and *Saperda vestita* (Cerambycidae). Environ. Entomol..

[38] Wang J.L., Nan X.N., Ren Z.Z., Ming J., Tang G.H. (2016). Diversity of intestinal bacteria communities from *Atrijuglans hetaohei* (Lepidoptera: Heliodinidae) and *Dichocrocis punctiferalis* (Lepidoptera: Pyralidae) larvae estimated by PCR-DGGE and T-RFLP analysis. Sci. Silvae Sin..

[39] Zhou H.Y., Sun B., Wu H.L., Hu X.M., Hao Y., Ye J.M. (2015). Research progress on function of insect’s gut microbiota and the microbial of *Bombyx mori*. N. Seric..

[40] Chen J.H., Zhou Y., Xia X.H., Zhao X.Y., Qiao H., Tan J.J., Hao D.J. (2021). Diversity and function of intestinal bacteria in adult *Monochamus alternatus* Hope (Coleoptera: Cerambycidae) fed indoors and outdoors. Acta Microbiol. Sin..

[41] Shukla S.P., Beran F. (2020). Gut microbiota degrades toxic isothiocyanates in a flea beetle pest. Mol. Ecol..

[42] Wang D.H., Wu W.J., Fu Y.G. (2012). Bacterial community in *Aleurodicus dispersus* (Hemiptera: Aleyrodidae) estimated by PCR-DGGE and 16S rRNA gene library analysis. Acta Entomol. Sin..

[43] Rowley S.M., Raven R.J., McGraw E.M. (2004). *Wolbachia pipientis* in Australian spiders. Curr. Microbiol..

[44] Hartelt K., Oehme R., Frank H., Brockmann S.O., Hassler D., Kimmig P. (2004). Pathogens and symbionts in ticks: Prevalence of *Anaplasma phagocytophilum* (*Ehrlichia* sp.), *Wolbachia* sp., *Rickettsia* sp., and *Babesia* sp. in southern Germany. Int. J. Med. Microbiol..

[45] Jiggins F.M. (2002). The rate of recombination in *Wolbachia* bacteria. Mol. Biol. Evol..

[46] Ros V.I.D., Breeuwer J.A.J., Menken S.B.J. (2008). Origins of asexuality in Bryobia mites (Acari: Tetranychidae). BMC Evol. Biol..

[47] Hilgenboecker K., Hammerstein P., Schlattmann P., Telschow A., Werren J.H. (2008). How many species are infected with *Wolbachia*?—A statistical analysis of current data. FEMS Microbiol. Lett..

[48] Werren J.H., Baldo L., Clark M.E. (2008). *Wolbachia*: Master manipulators of invertebrate biology. Nat. Rev. Microbiol..

[49] López-Hernández M.G., Rincón-Rosales R., Rincón-Molina C.I., Manzano-Gómez L.A., Gen-Jiménez A., Maldonado-Gómez J.C., Rincón-Molina F.A. (2025). Diversity and functional potential of gut bacteria associated with the insect *Arsenura armida* (Lepidoptera: Saturniidae). Insects.

[50] Suen G., Scott J.J., Aylward F.O., Adams S.M., Tringe S.G., Pinto-Tomás A.A., Foster C.E., Pauly M., Weimer P.J., Barry K.W. (2010). An insect herbivore microbiome with high plant biomass-degrading capacity. PLoS Genet..

[51] Jones R.T., Sanchez L.G., Fierer N. (2013). A cross-taxon analysis of insect-associated bacterial diversity. PLoS ONE.

